# Association of federal poverty level with healthcare expenditures among opioids users in the United States (2008–2019): a serial cross-sectional study

**DOI:** 10.1186/s12939-025-02413-6

**Published:** 2025-02-24

**Authors:** Mark Bounthavong, Kangho Suh, Aryana Sepassi, Britney Stottlemyer, Patrick Spoutz, Laura Hart, Meng Li

**Affiliations:** 1 Skaggs School of Pharmacy & Pharmaceutical Sciences, University of California San Diego, 9255 Pharmacy Lane, MC 0657, La Jolla, CA 92093-0657 USA; 2 VA Health Economics Resource Center, VA Palo Alto Healthcare System, 795 Willow Road (152 MPD), Menlo Park, CA 94025 United States of America; 3 School of Pharmacy, University of Pittsburgh, 3501 Terrace St, Pittsburgh, PA 15261 USA; 4 School of Pharmacy & Pharmaceutical Sciences, University of California Irvine, 856 Health Sciences Road, Irvine, CA 92697 USA; 5 US Department of Veterans Affairs, Pharmacy Benefits Management, 500 West Fort Street, Boise, ID 83702-4501 USA; 6 Center for the Evaluation of Value and Risk in Health, Tufts Medical Center, 800 Washington St, Boston, MA 02111 USA; 7 Plein Center for Aging, University of Washington, 1959 NE Pacific Street, Box 357630, Seattle, WA 98195-7630 USA

**Keywords:** Healthcare expenditures, Healthcare consumption, Federal poverty level, Opioids, United States, Medical Expenditure Panel Survey, Costs, Utilization

## Abstract

**Background:**

Opioid users across federal poverty levels have varying healthcare consumption, which could influence public health policies to address the opioid crisis. To better understand this relationship, we evaluated the associations between federal poverty level (FPL) with healthcare costs and utilizations among adult opioid users in the United States (US).

**Methods:**

A serial cross-sectional study using pooled data (2008–2019) from the Medical Expenditure Panel Survey (MEPS) was used to evaluate the association between FPL with healthcare expenditures among a representative sample of the US adult population with > = 1 opioid prescription. FPL was defined as Poor/Near Poor-Income, Low-Income, Middle-Income, and High-Income. Healthcare expenditures included costs and resource utilization. Survey weights were applied to generate standard errors for the representative sample of the US population. Generalized linear models were constructed to evaluate the association between FPL and healthcare expenditures adjusting for confounders. FPL groups were stratified by insurance coverage, frequency of opioid prescriptions filled, and pain level to evaluate their impact on healthcare expenditures.

**Results:**

Total weighted sample was 27,289,263 respondents; 21.6% in Poor/Near Poor-Income, 14.9% in Low-Income, 28.6% in Middle-Income, and 34.9% in High-Income groups. The average annual increase in total healthcare costs for the Poor/Near Poor-Income group was $451 (95% CI: $142-$761), $275 (95% CI: $48-$502) for the Low-Income group, $640 (95% CI: $447-$834) for the Middle-Income group, and $618 (95% CI: $360-$877) for the High-Income group. Between-group comparisons yielded significant increases in average annual total healthcare costs for Middle- and High-Income groups versus Low-Income group; significant increases in average annual emergency room costs between Middle- versus Low-Income groups, and significant increases in average annual inpatient costs between Middle-Income versus Poor/Near Poor- and Low-Income groups. Stratified analyses yielded several significant increases in average annual costs and expenditures. However, no differences were reported for respondents who were uninsured across FPL groups.

**Conclusions:**

Respondents across FPL groups consumed healthcare at various rates, particularly when stratified by insurance coverage, frequency of opioid prescriptions filled, and pain level. FPL plays an important role in healthcare consumption, but further research is needed to understand these mechanisms and their impact on the opioid crisis.

**Supplementary Information:**

The online version contains supplementary material available at 10.1186/s12939-025-02413-6.

## Introduction

Opioid-related overdoses and deaths have been increasing substantially in the United States (US) resulting in the US Department of Health and Human Services Acting Secretary to declare a public health emergency [1]. According to the US National Center on Health Statistics in June 2024, over 70,000 individuals have died from an opioid-related overdose, a 131% increase from June 2015 [2]. The Stanford–Lancet Commission on the North American Opioid Crisis identified several factors responsible for this public health emergency [3]. These factors include an unregulated opioid drug market, availability of cheaper alternative illicit drugs (e.g., heroin), and introduction of exponentially more potent synthetic opioids (e.g., fentanyl) [3]. Current public health strategies to address the opioid crisis emphasize interventions such as statewide prescription drug monitoring program (PDMP) to reduce inappropriate opioid prescribing, harm reduction treatment (e.g., naloxone) to reverse opioid-related overdose [4–9], pharmaceutical treatment (e.g., methadone, naltrexone, and buprenorphine) to treat opioid use disorder [10–16], cognitive behavioral therapy and psychosocial support to address addiction and misuse [17, 18], and optimal pain management [19, 20]. Although these efforts to curb the crisis have resulted in a decline from a peak of over 86,000 deaths in June 2023 [2], the opioid crisis has had a tremendous impact on the economic burden on society, which was estimated to be approximately $1.02 trillion [21].

According to the Grossman Model of Health Demand, individuals will choose to invest their time into the production of health (versus other activities, such as work and leisure) to optimally maximize their utility function subject to their constraints (health stock, time, and income) [22]. Awiti expands on this model with the Poverty and Health Care Demand framework where an individual’s predisposing factors (e.g., age, sex, education) and illness level can impact their poverty status thereby affecting the type of health care they access, which ultimately impact their overall health status (Appendix 1) [23]. Predisposing factors are based on the Andersen-Newman model of social determinants of healthcare consumption, which includes individual factors that can impact poverty such as insurance coverage and illness level (e.g., pain) [24, 25]. Therefore, addressing poverty and its associated interactions with other individual-level factors could be vitally important strategy in optimizing healthcare consumption and improving health status [26–32].

By understanding the complex association between poverty and its interaction with individual-level factors with healthcare expenditure, decision makers may be better informed to develop optimal policies to address the opioid crisis. Therefore, we sought to investigate the association between federal poverty level (FPL) and its interactions with insurance coverage, frequency of opioid fills, and self-reported pain level with healthcare expenditures among adult opioid users in the US using a representative sample from the Agency for Healthcare Research and Quality (AHRQ) Medical Expenditure Panel Survey (MEPS) between 2008 and 2019. We begin by evaluating the association between FPL with healthcare expenditures. Then, we stratified these findings across insurance coverage, frequency of opioid fills, and pain level to explore their influence on healthcare expenditures. We conclude by summarizing our findings and making recommendations for future policies to address the opioid crisis.

## Materials and methods

### Study design

We conducted a serial cross-sectional study using pooled data (2008 to 2019) from the AHRQ MEPS to evaluate the association between FPL and healthcare expenditures among respondents with > = 1 opioid prescriptions filled in the US. We adhered to the STrengthening the Reporting of OBservational studies in Epidemiology (STROBE) guidelines for the reporting of observational studies (Appendix 2) [33]. The primary aim evaluated the association between FPL groups and healthcare expenditures. Secondary aims evaluated the association between FPL and healthcare expenditures stratified by insurance coverage, frequency of opioid fills, and self-reported pain level.

### Sample

The analytic sample was pooled from household respondents between 2008 and 2019, which was based on the subsample of the National Health Interview Survey households, a nationally representative sample of the non-institutionalized US population [34]. We included adult respondents (> = 18 years old) who had > = 1 outpatient opioid prescription filled that was defined as a narcotic analgesic (including tramadol) or narcotic analgesic combination using the Cerner Multum Lexicon therapeutic class codes (Appendix 3). We included respondents who had a methadone or buprenorphine prescription filled for pain management (Appendix 3). Although methadone is indicated for opioid use disorder treatment, we assumed that outpatient prescription for methadone was indicated for pain. Methadone for opioid use disorder treatment is generally administered in an opioid treatment program facility, reducing the likelihood for outpatient methadone prescription. Additionally, we assumed that buprenorphine prescriptions filled were indicated for pain rather than opioid use disorder treatment based on the specific brand. For instance, Suboxone® and Sublocade® were FDA approved for treatment of opioid use disorder and were not considered opioid-related prescription for pain. Hence, respondents with a buprenorphine prescription for opioid use disorder treatment were excluded. Further, respondents were excluded if they reported having a diagnosis of cancer.

### Data source

MEPS is a set of large-scale surveys of US households and their medical providers [34]. MEPS collects data on the consumption of healthcare services such as the costs and the number of specific services from households drawn from a nationally representative subsample of the National Health Interview Survey. The Household Component of MEPS gathers data on the respondent’s demographics, medical conditions, and healthcare-related utilization and expenditures. Analytic weights are given to each respondent to account for the complex survey design, which are then used to estimate standard errors for the nationally representative population [35].

### Variables

The main variable of interest was FPL, which was categorized as Poor/Near Poor-Income (< 125% poverty line), Low-Income (125%-199% poverty line), Middle-Income (200%-399% poverty line), and High-Income (> = 400% poverty line). FPL is a measure of income developed by the Department of Health and Human Services that takes into account the minimum income an individual or family needs for food, clothing, shelter, and other necessities over the course of a year [36]. FPL is updated annually by DHHS and adjusted for inflation (Appendix 4) [37]. FPL is often used to determine whether individuals or families qualify for certain federal aid programs, such as Medicaid.

Healthcare expenditures were based on the MEPS Household Component and Medical Provider Component and included data on costs (total, office-based, outpatient, emergency room, inpatient, and prescription) and utilization (office-based visits, outpatient visits, emergency room visits, inpatient visits, hospitalization nights, and prescription fills) [38]. Total expenditures included costs and utilizations associated with the office-based, outpatient, emergency room, inpatient, prescription, and other expenditures. Office-based expenditures included costs and utilizations associated with the doctor’s office, medical clinic, or managed care plan center. Outpatient expenditures included costs and utilizations associated with the hospital outpatient department (e.g., services received at a hospital but do not require overnight hospitalization). Emergency room expenditures included costs and utilizations associated with the hospital emergency room. Inpatient expenditures include costs and utilizations associated with hospital overnight hospitalizations. Prescription expenditures included costs and utilizations associated with any prescription drugs ordered by a licensed healthcare professional for a pharmacist fill. Healthcare costs were based on payments rather than charges, including those made by private and public insurance, out-of-pocket, and other sources. Pooled costs (US dollars, $US) over the years were adjusted for inflation based on the 2023 Personal Consumption Expenditure (PCE) Health Index as recommended by AHRQ MEPS (Appendix 5) [39, 40]. Utilization was based on the counts of the number of medical-related events.

Additional variables included age category (18–24 years, 25–44 years, 45–64 years, and 65 + years), sex (male, female), race (White, Black, American Indian/Alaskan Native, Asian/Pacific Islander, and Multiple), ethnicity (Hispanic and Non-Hispanic), marital status (Never married/Unknown, Married, Widowed, and Divorced), education (No degree, high school equivalent, Associate degree, Bachelor degree, Master/Doctoral degree, and Unknown), region (Northwest, Midwest, South, and West), comorbidities (hypertension, coronary heart disease, angina, myocardial infarction, other heart diagnosis, stroke, high cholesterol, diabetes, and arthritis), and mental health illnesses (e.g., substance-related, schizophrenia or schizoaffective, mood, and anxiety). Comorbidities were defined using MEPS priority conditions, and mental health illnesses were defined using Clinical Classification Codes (Appendix 6). Substance-related illnesses included alcohol, tobacco, opioids, and others substance disorders defined by the Clinical Classification Codes (Appendix 6).

For the stratified analysis, respondents were categorized based on their insurance coverage, frequency of opioid fills, and pain level. Insurance coverage was categorized as Any private, Public, and Uninsured [41]. Private health insurance included non-public health insurance coverage including Medigap coverage; single-service plans such as dental, vision, or prescription plans were not included. Public health insurance was defined as not having Private insurance and coverage with Medicare, Medicaid, TRICARE, or other public hospital and physician coverage. Additionally, respondents who filled a prescription opioid during the year were categorized as “Any use” and “Frequent use.” “Any use” was defined as having between 1 to 3 opioid prescriptions filled during the year, and “Frequent use” was defined as having 4 or more opioid prescriptions filled during the year [42]. Further, respondents were categorized by their pain level based on the self-administered questionnaire item (“*During past 4 weeks, pain interfered with normal work outside the home and housework*”) [41]. Pain level was categorized as “Not at all,” “A little bit,” “Moderately,” “Quite a bit,” “Extremely,” and “Unknown/Refused/Not applicable.”

### Data analysis

Survey weights were applied to the pooled data using Stata’s set of svy commands and MEPS recommendations to generate results that would be representative of the US population [43, 44]. Descriptive analyses on baseline characteristics across the FPL groups were performed, and the means with standard deviation (SD) and frequencies with proportions were provided for continuous and categorical variables, respectively. Baseline comparisons were performed using one-way analysis of variance for continuous variables and chi square test for categorical variables.

For the primary aim, generalized linear models (GLM) using gamma distribution for healthcare costs and negative binomial distribution for healthcare utilization were constructed to evaluate the association between FPL groups and healthcare expenditures adjusting for baseline demographics. Interaction terms between FPL group with insurance coverage, frequency of opioid prescription filled, reported pain level, and year were added for the trend and stratified analyses. The regression models controlled for age category, sex, race, ethnicity, marital status, education, region, comorbidities (hypertension, coronary heart disease, angina, myocardial infarction, other heart diagnosis, stroke, hypercholesterolemia, cancer, diabetes, and arthritis), and mental health illnesses (substance-related, schizophrenia/other psychotic disorder, mood, and anxiety). Results were presented as the average annual change in healthcare costs and utilization (slope) with their corresponding 95% confidence intervals (CI). For the comparisons between the FPL groups, the difference in the average annual change in healthcare costs and utilization with their corresponding 95% CI were presented. Goodness of fit tests included the Pearson correlation of the predicted and residual values, Pregibon’s link test, and modified Hosmer–Lemeshow test [45].

For the secondary aims, the average annual change in healthcare costs and utilization for each FPL group were stratified based on insurance coverage, frequency of opioid prescriptions filled, and pain level. Results were presented as the average annual change in healthcare costs and utilization with their corresponding 95% CI.

The statistical threshold was set at a two-tailed alpha of less than 5%, and all analyses were performed using Stata SE version 18 (Stata Corp, LLC, College Station, TX).

## Results

The total weighted sample of respondents with > = 1 opioid prescription filled in the US was 27,289,263; 29.9% in the Poor/Near Poor-Income, 16.8% in the Low-Income, 27.7% in the Middle-Income, and 25.7% in the High-Income groups (Table 1). Respondents were, on average, 48.9 years old, mostly female (59.1%), White (80.6%), non-Hispanic (89.1%), and Married (51.4%). Due to the large, weighted sample, all baseline demographic comparisons were significant across the FPL groups; however, several were considered meaningfully different. Compared to the High-Income Group, the Low-Income Group had more females (65.4% vs. 55.3%), minorities (28.1% vs 13.8%), Hispanics (14.2% vs. 7.1%), divorcees (30.6% vs 11.0%), and respondents with no degree (28.9% vs. 5.6%). Notably, respondents who were Poor/Near Poor-Income had a lower proportion with Private insurance (23.5%) compared to the Low-Income (46.8%), Middle-Income (73.6%), and High-Income (89.0%) groups. Additionally, respondents in the Poor/Near Poor-Income group had a higher proportion (39.9%) categorized as Frequent users of opioids (> = 4 opioid prescriptions filled) than the High-Income group (20.0%). Further, respondents in the Poor/Near Poor-Income group had a higher proportion (16.9%) categorized with “Extremely” pain level that interfered with their normal work and housework than the High-Income group (5.1%). No differences in mental health illnesses were reported across the FPL groups.

**Table 1:**
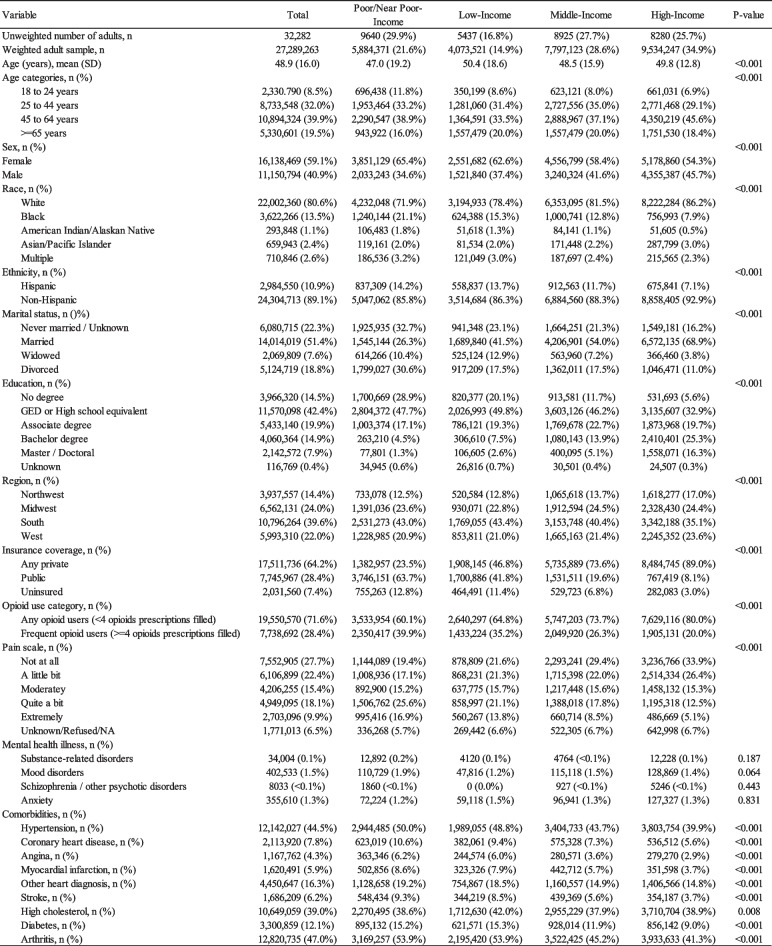
Characteristics of respondents who filled an opioid prescription by federal poverty level (FPL) groups

*SD* standard deviation, *NA* not applicable

### Unadjusted results

**Fig. 1:**
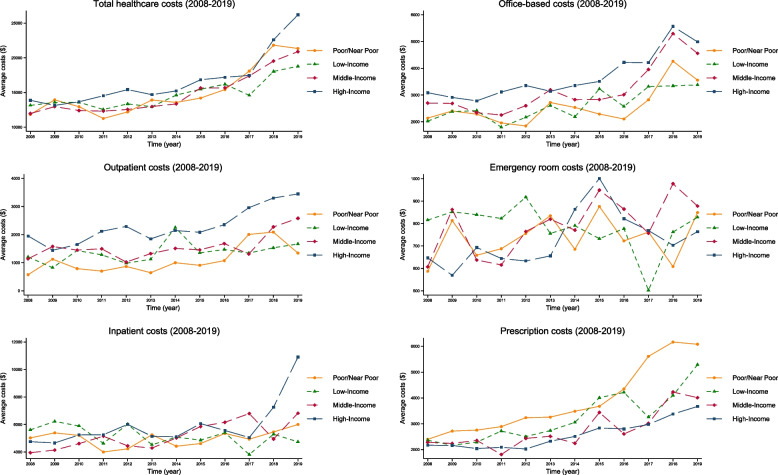
Healthcare cost trends across federal poverty levels among respondents who had reporting filling an opioid prescription (2008–2019)

**Fig. 2:**
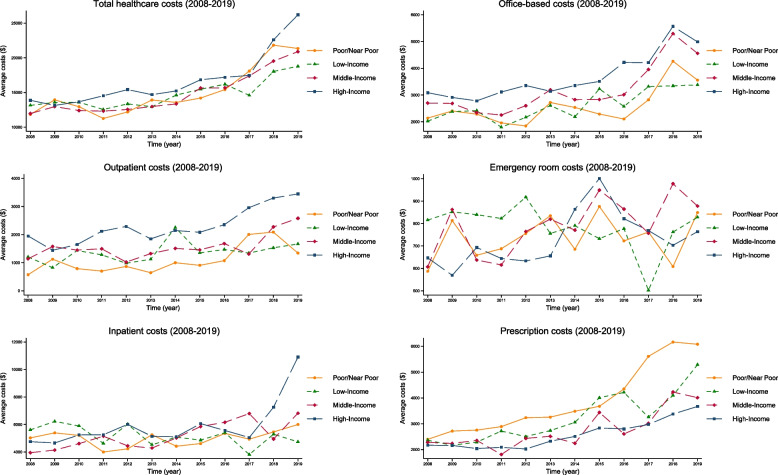
Healthcare resource utilization trends across federal poverty levels among respondents who had reporting filling an opioid prescription (2008–2019)

Between 2008 and 2019, the number respondents with > = 1 opioid prescriptions filled decreased by approximately 31% (Appendix 7). The average annual total healthcare costs were $15,714 for respondents with an opioid prescription; inpatient costs made up a large part of the total healthcare costs ($5382) followed by office-based visit costs ($3188), prescription costs ($3072), outpatient visit costs ($1823), and emergency room visit costs ($766) (Appendix 8). Significant differences were reported for total healthcare costs, office-based visit costs, outpatient visit costs, and prescription costs across the FPL groups. On average, respondents had 11.97 office-based visits, 1.30 outpatient visits, 0.58 emergency room visits, 0.31 hospital discharges, 1.36 hospital night stays, and 27.43 prescriptions filled per year. Significant differences were reported for the number of office-based visits, outpatient visits, emergency room visits, hospital discharges, nights of hospitalization, and prescription fills. Figures 1 and 2 illustrate the trends for all healthcare expenditures (costs and utilizations) categories by FPL groups.

### Regression results

In the GLM results, significant increases in average annual total healthcare costs were reported across the FPL groups (Table 2). The average annual increase in total healthcare costs for the Poor/Near Poor-Income group was $451 (95% CI: 142, 761), $275 (95% CI: 48, 502) for the Low-Income group, $640 (95% CI: 447, 834) for the Middle-Income group, and $618 (95% CI: 360, 877) for the High-Income group. Significant annual increases in office-based costs and visits, prescription costs, outpatient visits, and emergency room visits were reported for all FPL groups.

**Table 2:**
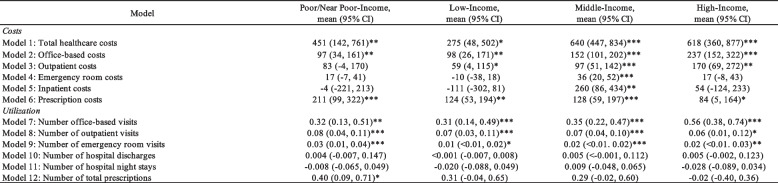
Average annual change in healthcare expenditures by federal poverty level (FPL) groups among respondents with > = 1 opioid prescription filled

*P<0.05; **P<0.01, ***P<.0.001

CI confidence interval

Covariates used in the regression model included age category, sex, race, ethnicity, marital status, education, region, insurance coverage, comorbities (hypertension, coronary heart disease, angina, myocardial infarction, other heart diagnosis, stroke, high cholesterol, diabetes, and arthritis), and behavioral disorders

Comparisons between the FPL groups yield several significant differences in the average annual changes in healthcare expenditures (Table 3). Respondents in the Middle-Income and High-Income groups had a greater annual increase in total healthcare costs compared to the Low-Income group (+ $365 and + $343, respectively). Respondents in the High-Income group had greater average annual increases in office-based costs compared to Poor/Near Poor-Income (+ $140) and Low-Income (+ $139) groups. Respondents in the Middle-Income group had greater average annual increases in emergency room and inpatient costs compared to the Low-Income group (+ $46 and + $370, respectively). Respondents in the Middle-Income group had significantly greater average annual inpatient costs compared to the Poor/Near Poor-Income group (+ $264). No differences in healthcare resource utilization were reported for all FPL group comparisons.

**Table 3:**
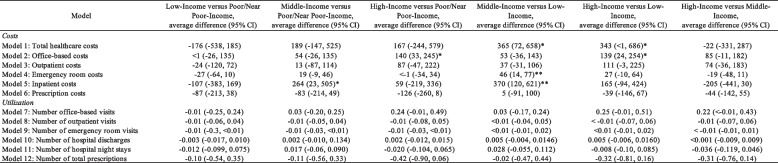
Average difference in annual change by federal poverty level (FPL) group on healthcare expenditures among respondents with > = 1 opioid prescription filled

*P<0.05; **P<0.01, ***P<.0.001

CI confidence interval

Covariates used in the regression model included age category, sex, race, ethnicity, marital status, education, region, insurance coverage, frequency of opioid prescription filled, pain scale, comorbities (hypertension, coronary heart disease, angina, myocardial infarction, other heart diagnosis, stroke, high cholesterol, diabetes, and arthritis), and behavioral disorders

### Stratified analyses

The weighted numbers and proportions of respondents by FPL group, insurance coverage, frequency of opioid prescription filled, and pain level are available in Appendix 9.

#### Insurance coverage

Between 2008 and 2019, the weighted number of respondents with > = 1 opioid prescription decreased by 38% if they had Private insurance, increased by 16% if they had Public insurance, and decreased by 84% if they were Uninsured (Appendix 9). In the stratified analysis, FPL groups stratified by insurance coverage yielded significant differences across all healthcare expenditure categories between 2008 and 2019 (Appendix 10). Respondents with Private insurance and in the High-Income and Middle-Income groups had significant increases in average annual total healthcare costs (+ $882 and + $885, respectively); whereas respondents in the Poor/Near Poor-Income and Low-Income groups with Public insurance had significant increases in average annual total healthcare costs (+ $636 and + $397, respectively). There were other significant increases in healthcare cost and resource utilizations across FPL groups stratified by insurance coverage, which are presented in Appendix 10. For instance, respondents across all FPL groups with Public insurance had significant increases in average annual office-based visits costs. However, respondents who were uninsured did not have significant increases in healthcare-related costs and utilizations for any of the FPL groups.

#### Frequency of opioid prescriptions filled

Between 2008 and 2019, the weighted number of respondents with > = 1 opioid prescription decreased by 34% if they had < 4 opioid prescriptions filled and decreased by 21% if they had 4 or more opioid prescriptions filled (Appendix 9). In the stratified analysis, respondents who were “Frequent” users of opioids and in the Low-Income and Middle-Income groups had significant increases in average annual total healthcare costs (+ $397 and + $474, respectively). Respondents who were “Any” users of opioids and in the Poor/Near Poor-Income, Middle-Income, and High-Income groups had significant increases in average annual total healthcare costs (+ $437, + $731, and + $751, respectively). Moreover, respondents categorized as “Any” users and “Frequent” users of opioids had significant increases in other healthcare cost and utilization categories (Appendix 10). For instance, respondents who were categorized as “Any” opioid user had a significant increase in average annual prescription costs for all FPL groups; however, respondents categorized as “Frequent” users had significant increases in average prescription costs for the Poor/Near Poor-Income, Low-Income, and Middle-Income groups.

#### Pain scale

Between 2008 and 2019, the weighted number of respondents with > = 1 opioid prescription decreased by 29% if they reported no pain (“Not at all”), decreased by 44% if they reported “A little” pain, decreased by 45% if they reported “Moderate” pain, decreased by 37% if they reported “Quite a bit” of pain, decreased by 44% if they reported extreme (“Extremely”) pain, and increased by 53% if their pain was unknown (Appendix 9). In the stratified analysis, respondents in the Middle-Income and High-Income groups with any pain level response had significant increases in average annual total healthcare costs (Appendix 10). No significant increase in the average annual healthcare costs was reported for respondents in the Poor/Near Poor-Income and Low-Income groups across all pain levels (except for the Unknown/Refused/NA pain scale category). Notably, respondents in the Poor/Near Poor-Income group had significantly greater increase in average annual number of emergency room visits for all pain levels (except for “A little bit”). Additionally, respondents in the Middle-Income and High-Income groups had significantly greater increase in average annual office-based visits costs and outpatient visits costs, which align with significant increases in the average number of office-based visits and outpatient visits.

## Discussion

Among respondents with > = 1 opioid prescription filled in a calendar year, average annual increases in healthcare expenditures varied by FPL groups stratified by insurance coverage, frequency of opioid prescriptions filled, and reported pain level. Significant differences in average annual changes in total healthcare costs were reported for comparisons between the Middle-Income and Low-Income groups and between the High-Income and Low-Income groups. Other pairwise differences were reported for office-based costs between the High-Income and Poor/Near Poor-Income groups and between the High-Income and Low-Income groups, emergency room costs between the Middle-Income and Low-Income groups, and inpatient costs between the Middle-Income and Poor/Near Poor-Income groups and between the Middle-Income and Low-Income groups. No significant differences were reported for the resource utilizations endpoints.

While the literature strongly links poverty with health outcomes [26–31, 46, 47], the interaction between FPL with insurance coverage, frequency of opioid prescriptions filled, and pain level complicates efforts for policymakers to develop effective policy interventions to address the opioid crisis. Those with more wealth have better health and those with less wealth have lower health. Impoverished individuals are also vulnerable to the mechanism that cause income disparity such that they find themselves invariably entangled in the revolving door of the health-poverty trap [48, 49]. Individuals with Low-Income often cannot afford essential treatment for their opioid addiction due to a lack of employer-sponsored health benefits, which can have downstream consequences such as increased morbidity and mortality. Whereas, individuals with High-Income have greater use of preventative care services, which result in lower usage of acute emergency or inpatient care services [47]. In our findings, we observed that respondents in the High-Income group had greater average annual increases in total healthcare costs and office-based visits compared to respondents in the Low-Income group suggesting potential use of preventative services. However, we did not observe the Poor/Near Poor-Income group consume more inpatient services when compared to other FPL groups, which we would have expected with impoverished individuals experiencing acute health events.

Policy makers are in a unique position to target individual social determinants of health that are directly or indirectly related to poverty. Expanding access to public insurance coverage (e.g., Medicaid) can help to alleviate the economic burden of impoverished opioid users. For instance, Sommers and colleagues reported that Medicaid expansion in Kentucky and use of Medicaid funds to purchase private insurance in Arkansas increased outpatient utilization, preventative services, and self-reported health among Low-Income individuals compared to Texas, which did not expand Medicaid coverage [50]. However, this may not be enough for impoverished opioid users who require additional treatments such as cognitive behavioral therapy, harm reduction therapy, and medication for opioid use disorder. Comprehensive policies addressing the systematic consequences of poverty are needed, particularly for opioid users. The threat of homelessness, lack of basic essential needs such as healthy groceries, and protected time to seek and maintain care are often neglected by policy makers. Consequently, impoverished individuals are reluctant to make choices that would improve their health due to conflicting financial priorities [29]. Ultimately, to address the opioid crisis, society and policy makers will need to make a concerted effort to implement strategies that target factors associated with poverty in addition to providing access to harm reduction treatment, improved pain management, and treatments for opioid use disorder.

Our study is not without limitations. We espoused the need to provide a set of policies to reduce poverty to improve the health outcomes of opioid users. However, we did not capture data on incarcerations, a common occurrence among individuals with substance use disorders. Incarcerations destabilizes the financial situation of an individual and may result in catastrophic economic burden which perpetuate the health-poverty trap [51]. Consequently, further research should make a greater effort to capture data on incarcerations across the FPL groups [52]. Additionally, our study focused on identifying respondents with an opioid prescription, but we were unable to identify illicit opioid users, which would have an impact on their healthcare expenditures. Previous studies have reported significant economic burden associated with illicit opioid users, which has also been linked with incarcerations [53–55]. Moreover, the cross-sectional design of this study prevents establishing a temporal relationship between opioid use and healthcare expenditure. Hence, our results should not be interpreted as a causal relationship; rather, we can only conclude that there is a statistical association. Next, the interactions between race, income, and health status have been associated with insurance coverage, which can impact health consumption and status. Among a representative sample of the US population, low-income minorities in bad health and low-income White individuals with bad health have reduced odds (81% and 48% reduction, respectively) of having health insurance coverage compared to high-income White individuals in good health [56]. To account for this we included race, poverty, insurance coverage, and pain level as covariates in our regression model. Further, we were unable to determine if our respondents were first-time opioid users or chronic users, which would invariably impact their healthcare expenditures. Chronic opioid users have been associated with higher healthcare expenditures compared to non-users [57]. Morphine milligram equivalents would have been a useful measure of chronic, high-dose opioid use, but we were unable to estimate this for all our respondents. Instead, we adopted MEPS definition of “Frequent” users of opioids (> = 4 opioid prescriptions filled) in our stratified analysis to as a proxy for chronic opioid users, but the results were mixed [42]. Therefore, it is unclear whether our analysis is representative of chronic, high-dose opioid users.

## Conclusions

Patterns in healthcare expenditures varied across FPL groups, particularly when stratified by insurance coverage, frequency of opioid prescriptions filled, and pain level. Although FPL plays an important role in the pathway towards health consumption and status, it is further complicated by other individual social determinants of health. Further research is needed to understand the mechanisms that lead to these differences in healthcare consumption and to assist health policy makers to design and invest in strategies to prevent further exacerbations of the opioid crisis.

## Supplementary Information

Supplementary Material 1.

## Data Availability

The datasets used and/or analyzed during the current study are publicly available from the Agency for Healthcare Research and Quality (AHRQ) Medical Expenditure Panel Survey.
