# Supplementary material for: Association of federal poverty level with healthcare expenditures among opioids users in the United States (2008–2019): a serial cross-sectional study
Source: Int J Equity Health. 2025 Feb 24;24:51. doi: 10.1186/s12939-025-02413-6 (PMC11849152; doi:10.1186/s12939-025-02413-6)
Supplement: Supplementary file 1 — Supplementary Material 1. [file 12939_2025_2413_MOESM1_ESM.docx]

**Appendices**

**Appendix 1. Poverty and Health Care Demand Framework.**


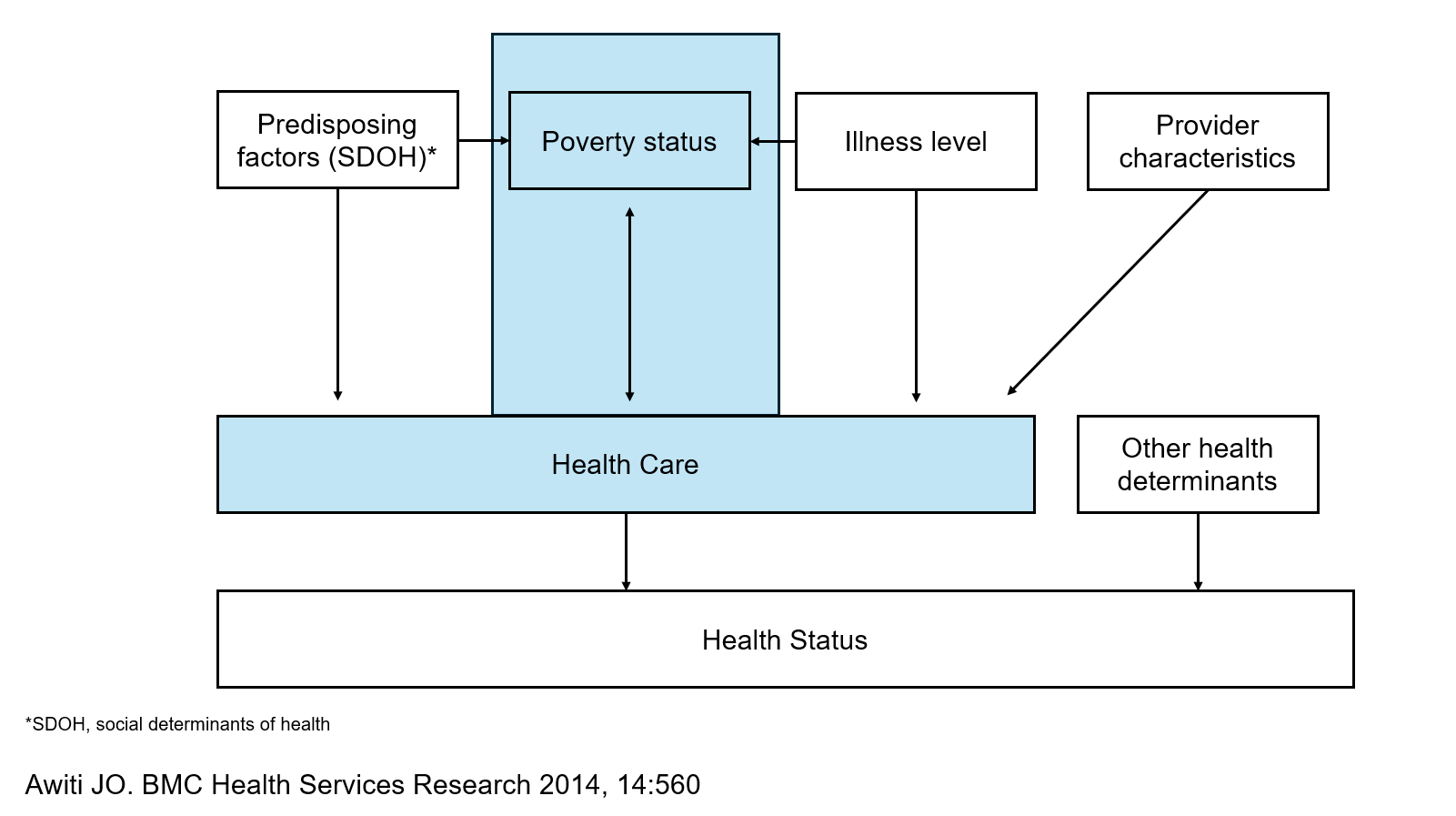


**Appendix 2**

**STROBE Statement—Checklist of items that should be included in reports of *cross-sectional studies***

***(These page numbers and location were based on the Word document submitted with the original submission.)***

|  | Item No | Recommendation | Page No |
| --- | --- | --- | --- |
| **Title and abstract** | 1 | (*a*) Indicate the study’s design with a commonly used term in the title or the abstract | 1 |
|  |  | (*b*) Provide in the abstract an informative and balanced summary of what was done and what was found | 5-6 |
| Introduction | | | |
| Background/rationale | 2 | Explain the scientific background and rationale for the investigation being reported | 8-9 |
| Objectives | 3 | State specific objectives, including any prespecified hypotheses | 9 |
| Methods | | | |
| Study design | 4 | Present key elements of study design early in the paper | 9 |
| Setting | 5 | Describe the setting, locations, and relevant dates, including periods of recruitment, exposure, follow-up, and data collection | 9 |
| Participants | 6 | (*a*) Give the eligibility criteria, and the sources and methods of selection of participants | 9-10 |
| Variables | 7 | Clearly define all outcomes, exposures, predictors, potential confounders, and effect modifiers. Give diagnostic criteria, if applicable | 10-12 |
| Data sources/ measurement | 8* | For each variable of interest, give sources of data and details of methods of assessment (measurement). Describe comparability of assessment methods if there is more than one group | 10 |
| Bias | 9 | Describe any efforts to address potential sources of bias | 13-14 |
| Study size | 10 | Explain how the study size was arrived at | NA |
| Quantitative variables | 11 | Explain how quantitative variables were handled in the analyses. If applicable, describe which groupings were chosen and why | 10-12 |
| Statistical methods | 12 | (*a*) Describe all statistical methods, including those used to control for confounding | 13-14 |
|  |  | (*b*) Describe any methods used to examine subgroups and interactions | 13-14 |
|  |  | (*c*) Explain how missing data were addressed | NA |
|  |  | (*d*) If applicable, describe analytical methods taking account of sampling strategy | 10, 13-14 |
|  |  | (*e*) Describe any sensitivity analyses | NA |
| Results | | | |
| Participants | 13* | (a) Report numbers of individuals at each stage of study—eg numbers potentially eligible, examined for eligibility, confirmed eligible, included in the study, completing follow-up, and analysed | 14 |
|  |  | (b) Give reasons for non-participation at each stage | NA |
|  |  | (c) Consider use of a flow diagram | NA |
| Descriptive data | 14* | (a) Give characteristics of study participants (eg demographic, clinical, social) and information on exposures and potential confounders | 14 |
|  |  | (b) Indicate number of participants with missing data for each variable of interest | NA |
| Outcome data | 15* | Report numbers of outcome events or summary measures | 14-17 |
| Main results | 16 | (*a*) Give unadjusted estimates and, if applicable, confounder-adjusted estimates and their precision (eg, 95% confidence interval). Make clear which confounders were adjusted for and why they were included | 14-15 |
|  |  | (*b*) Report category boundaries when continuous variables were categorized | NA |
|  |  | (*c*) If relevant, consider translating estimates of relative risk into absolute risk for a meaningful time period | NA |
| Other analyses | 17 | Report other analyses done—eg analyses of subgroups and interactions, and sensitivity analyses | 16-17 |
| Discussion | | | |
| Key results | 18 | Summarise key results with reference to study objectives | 18-20 |
| Limitations | 19 | Discuss limitations of the study, taking into account sources of potential bias or imprecision. Discuss both direction and magnitude of any potential bias | 20 |
| Interpretation | 20 | Give a cautious overall interpretation of results considering objectives, limitations, multiplicity of analyses, results from similar studies, and other relevant evidence | 18-20 |
| Generalisability | 21 | Discuss the generalisability (external validity) of the study results | 20 |
| Other information | | | |
| Funding | 22 | Give the source of funding and the role of the funders for the present study and, if applicable, for the original study on which the present article is based | NA |

*Give information separately for exposed and unexposed groups.

**Note:** An Explanation and Elaboration article discusses each checklist item and gives methodological background and published examples of transparent reporting. The STROBE checklist is best used in conjunction with this article (freely available on the Web sites of PLoS Medicine at http://www.plosmedicine.org/, Annals of Internal Medicine at http://www.annals.org/, and Epidemiology at http://www.epidem.com/). Information on the STROBE Initiative is available at www.strobe-statement.org.

**Appendix 3. Identifying opioid prescription fills**

We used the Cerner Multum Lexicon codes to identify narcotic prescriptions filled, which included a variety of opioid-related prescriptions. These variables included:

tc1s1_1 == 60 | tc1s1_1 == 191

We also used the Prescription Drug File. The RXDRGNAM variable included the generic of the drug. The RXNAME included both the generic and brand name of the drug.

We used a combination of the Cerner Multum Lexicon codes, RXDRGNAME, and RXNAME variables in MEPS Full-Year Consolidated Data File to identify the opioid prescription.

For buprenorphine, we had to identify the formulations that were FDA-approved for pain management rather than addiction treatment. We used the brand name of buprenorphine that was associated with pain management. These included Burenex®, Burans®, and Belbuca®. We assumed that other formulations of buprenorphine were for addiction treatment (e.g., Suboxone®).

Here is a sample Stata code for the 2008 Full-Year Consolidated Data File.

/* RXDRGNAM generic name of the drug most commonly used by prescribing physicians */

/* Tramadol was not a narcotic prior to 2024; thus, need to include it */

gen byte tramadol = strpos(rxname, "TRAMADOL") | strpos(rxname, "TRAMAD") > 0

/* Buprenorphine for pain should be included */

gen byte buprenorphine = strpos(rxname, "BUPRENORPHINE") | strpos(rxname, "BUPRENEX") | strpos(rxname, "BUTRANS") | strpos(rxname, "BELBUCA") > 0

***** Keep narcotic analgesics and narcotic combination analgesics (Note: include tramadol)

keep if tc1s1_1 == 60 | tc1s1_1 == 191 | tramadol == 1 | buprenorphine == 1

**Appendix 4. U.S. Department of Health and Human Services Poverty Line Threshold, 2008-2019.**

| Department of Health and Human Services Poverty Line Thresholds. | | | |
| --- | --- | --- | --- |
| Year | Single Person | Each additional person | Four-Person Family |
| 2024 | $15,060 | $5,380 | $31,200 |
| 2023 | $14,580 | $5,140 | $30,000 |
| 2022 | $13,590 | $4,720 | $27,750 |
| 2021 | $12,880 | $4,540 | $26,500 |
| 2020 | $12,760 | $4,480 | $26,200 |
| 2019 | $12,490 | $4,420 | $25,750 |
| 2018 | $12,140 | $4,320 | $25,100 |
| 2017 | $12,060 | $4,180 | $24,600 |
| 2016 | $11,880 | Varies* | $24,300 |
| 2015 | $11,770 | $4,160 | $24,250 |
| 2014 | $11,670 | $4,060 | $23,850 |
| 2013 | $11,490 | $4,020 | $23,550 |
| 2012 | $11,170 | $3,960 | $23,050 |
| 2011 | $10,890 | $3,820 | $22,350 |
| 2010 | $10,830 | $3,740 | $22,050 |
| 2009 | $10,830 | $3,740 | $22,050 |
| 2008 | $10,400 | $3,600 | $21,200 |
| * In 2016, the average difference between 1 additional person ranged between $4140 to $4160. | | | |

**Source:** [**https://aspe.hhs.gov/topics/poverty-economic-mobility/poverty-guidelines/prior-hhs-poverty-guidelines-federal-register-references**](https://aspe.hhs.gov/topics/poverty-economic-mobility/poverty-guidelines/prior-hhs-poverty-guidelines-federal-register-references)

**Appendix 5. Personal Consumption Expenditure (PCE) Health Total Price Index.**

| Personal Consumption Expenditures (PCE) Health Total Price Index | | |
| --- | --- | --- |
| Year | PCE-Health | Reference (2023) |
| 2008 | 85.121 | 1.328920008 |
| 2009 | 87.462 | 1.293350255 |
| 2010 | 89.740 | 1.260519278 |
| 2011 | 91.560 | 1.235463084 |
| 2012 | 93.374 | 1.211461435 |
| 2013 | 94.511 | 1.196887135 |
| 2014 | 95.828 | 1.180437868 |
| 2015 | 96.830 | 1.168222658 |
| 2016 | 98.349 | 1.150179463 |
| 2017 | 100.000 | 1.13119 |
| 2018 | 101.745 | 1.111789277 |
| 2019 | 103.247 | 1.095615369 |
| 2020 | 105.442 | 1.0728078 |
| 2021 | 107.571 | 1.051575239 |
| 2022 | 110.239 | 1.026125056 |
| 2023 | 113.119 | 1 |

**Source:** [**https://apps.bea.gov/histdatacore/histChildLevels.html?HMI=7&oldDiv=National%20Accounts**](https://apps.bea.gov/histdatacore/histChildLevels.html?HMI=7&oldDiv=National%20Accounts)

**Appendix 6. Codes used for comorbidities from MEPS data files.**

**Appendix 7. Survey-weighted number of respondents with >=1 opioid prescription filled.**

| Year | Total |
| --- | --- |
| 2008 | 2,327,528 |
| 2009 | 2,367,036 |
| 2010 | 2,403,900 |
| 2011 | 2,483,686 |
| 2012 | 2,401,288 |
| 2013 | 2,520,367 |
| 2014 | 2,621,176 |
| 2015 | 2,561,687 |
| 2016 | 2,115,825 |
| 2017 | 1,977,410 |
| 2018 | 1,896,052 |
| 2019 | 1,613,309 |

**
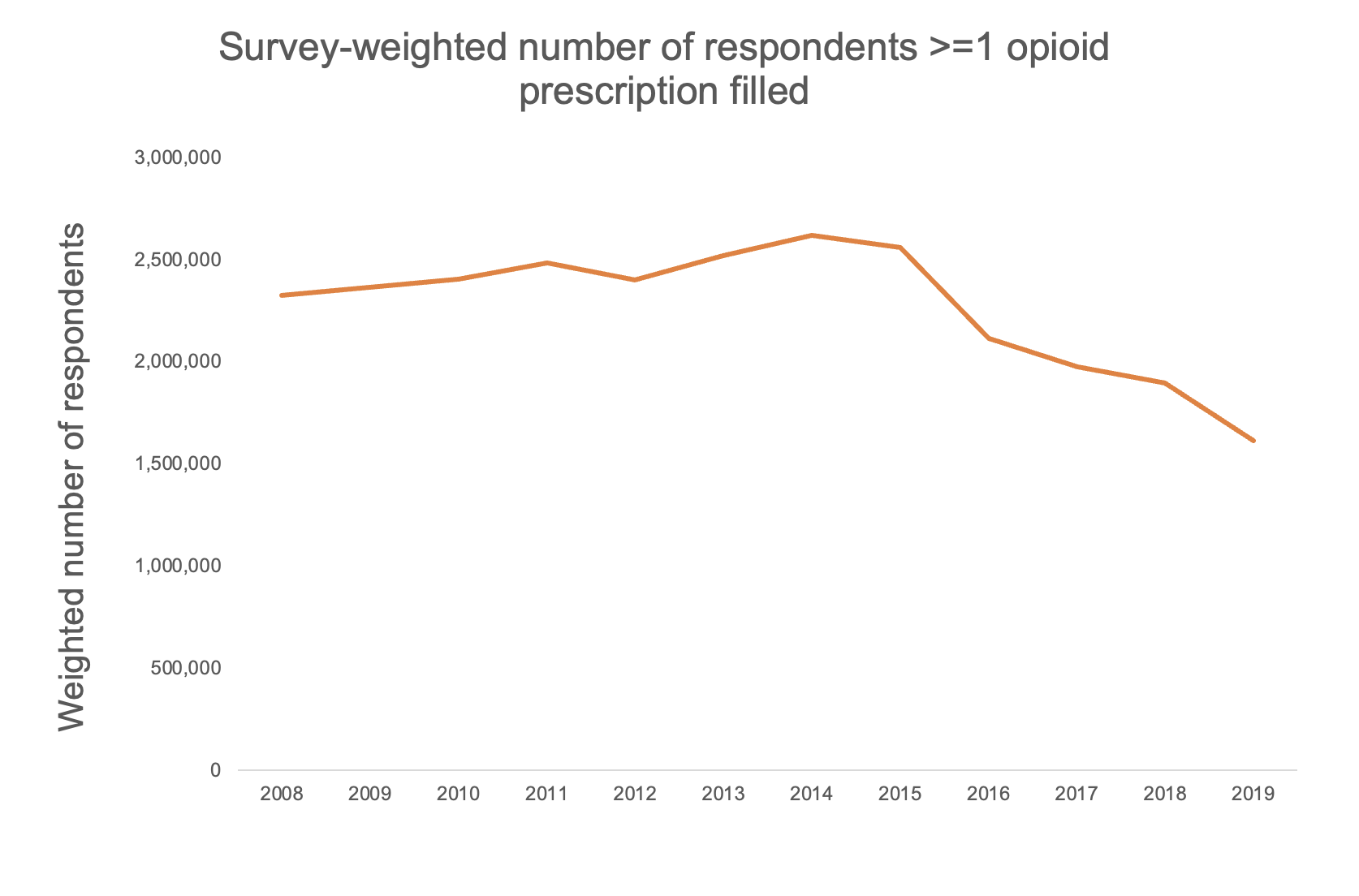
**

**Appendix 8. Healthcare expenditures across federal poverty levels among respondents who had reporting filling an opioid prescription, 2008-2019 (unadjusted)**

**Appendix 9. Number and proportion of respondents with 1 or more opioid prescription by subgroups.**

**Appendix 9A. Number/proportion of respondents with 1 or more opioid prescription by FPL category.**

| Weighted totals | |  |  |  |  |
| --- | --- | --- | --- | --- | --- |
| Year | Poor/Near poor | Low income | Middle income | High income | Total |
| 2008 | 441,000 | 381,849 | 676,349 | 828,330 | 2,327,528 |
| 2009 | 475,060 | 393,683 | 685,839 | 812,454 | 2,367,036 |
| 2010 | 555,502 | 346,432 | 693,337 | 808,628 | 2,403,900 |
| 2011 | 595,209 | 367,021 | 747,671 | 773,786 | 2,483,686 |
| 2012 | 556,005 | 354,916 | 720,865 | 769,502 | 2,401,288 |
| 2013 | 596,027 | 388,327 | 696,897 | 839,116 | 2,520,367 |
| 2014 | 621,842 | 355,017 | 726,371 | 917,947 | 2,621,176 |
| 2015 | 518,177 | 415,774 | 674,315 | 953,421 | 2,561,687 |
| 2016 | 421,629 | 293,512 | 593,072 | 807,612 | 2,115,825 |
| 2017 | 397,815 | 291,038 | 556,579 | 731,978 | 1,977,410 |
| 2018 | 393,064 | 261,829 | 546,391 | 694,767 | 1,896,052 |
| 2019 | 313,042 | 224,124 | 479,436 | 596,706 | 1,613,309 |


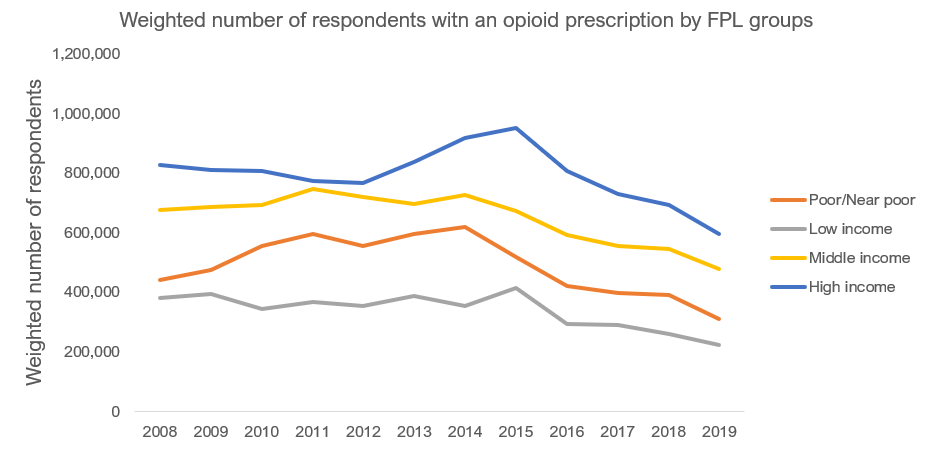


| Weighted proportions | |  |  |  |
| --- | --- | --- | --- | --- |
| Year | Poor/Near poor | Low income | Middle income | High income |
| 2008 | 0.189 | 0.164 | 0.291 | 0.356 |
| 2009 | 0.201 | 0.166 | 0.290 | 0.343 |
| 2010 | 0.231 | 0.144 | 0.288 | 0.336 |
| 2011 | 0.240 | 0.148 | 0.301 | 0.312 |
| 2012 | 0.232 | 0.148 | 0.300 | 0.320 |
| 2013 | 0.236 | 0.154 | 0.277 | 0.333 |
| 2014 | 0.237 | 0.135 | 0.277 | 0.350 |
| 2015 | 0.202 | 0.162 | 0.263 | 0.372 |
| 2016 | 0.199 | 0.139 | 0.280 | 0.382 |
| 2017 | 0.201 | 0.147 | 0.281 | 0.370 |
| 2018 | 0.207 | 0.138 | 0.288 | 0.366 |
| 2019 | 0.194 | 0.139 | 0.297 | 0.370 |


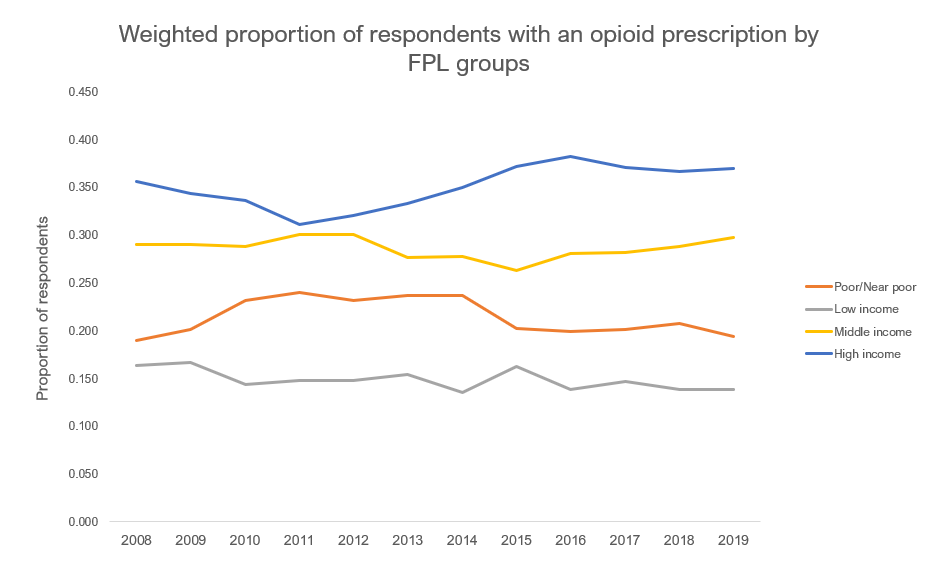


**Appendix 9B. Number/proportion of respondents with 1 or more opioid prescriptions by insurance coverage.**

| Weighted totals | | | | |
| --- | --- | --- | --- | --- |
| Year | Any private | Public | Uninsured | Total |
| 2008 | 1,583,110 | 511,105 | 233,313 | 2,327,528 |
| 2009 | 1,560,251 | 542,542 | 264,242 | 2,367,036 |
| 2010 | 1,523,253 | 639,450 | 241,197 | 2,403,900 |
| 2011 | 1,603,991 | 649,443 | 230,252 | 2,483,686 |
| 2012 | 1,513,071 | 635,191 | 253,026 | 2,401,288 |
| 2013 | 1,583,420 | 693,628 | 243,320 | 2,520,367 |
| 2014 | 1,664,423 | 800,628 | 156,125 | 2,621,176 |
| 2015 | 1,668,890 | 768,105 | 124,692 | 2,561,687 |
| 2016 | 1,367,816 | 644,228 | 103,781 | 2,115,825 |
| 2017 | 1,268,161 | 631,597 | 77,652 | 1,977,410 |
| 2018 | 1,193,108 | 637,311 | 65,632 | 1,896,052 |
| 2019 | 982,242 | 592,739 | 38,327 | 1,613,309 |


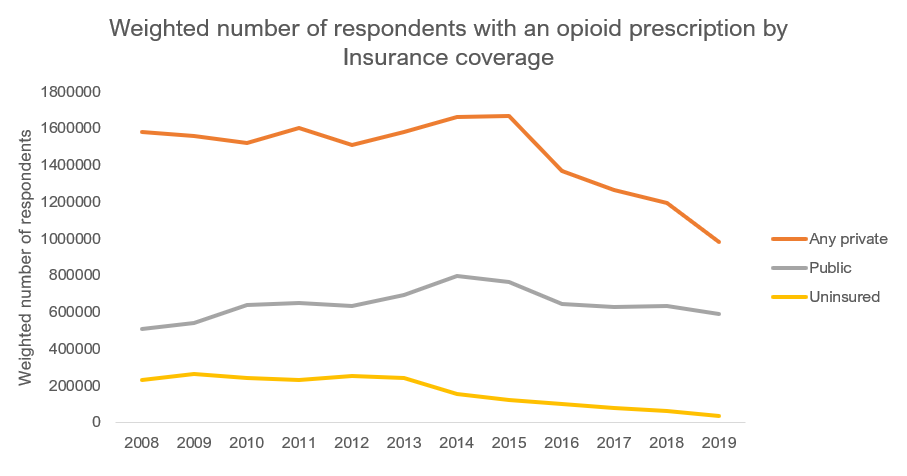


| Weighted proportions | |  |  |
| --- | --- | --- | --- |
| Year | Any private | Public | Uninsured |
| 2008 | 0.680 | 0.220 | 0.100 |
| 2009 | 0.659 | 0.229 | 0.112 |
| 2010 | 0.634 | 0.266 | 0.100 |
| 2011 | 0.646 | 0.261 | 0.093 |
| 2012 | 0.630 | 0.265 | 0.105 |
| 2013 | 0.628 | 0.275 | 0.097 |
| 2014 | 0.635 | 0.305 | 0.060 |
| 2015 | 0.651 | 0.300 | 0.049 |
| 2016 | 0.646 | 0.304 | 0.049 |
| 2017 | 0.641 | 0.319 | 0.039 |
| 2018 | 0.629 | 0.336 | 0.035 |
| 2019 | 0.609 | 0.367 | 0.024 |


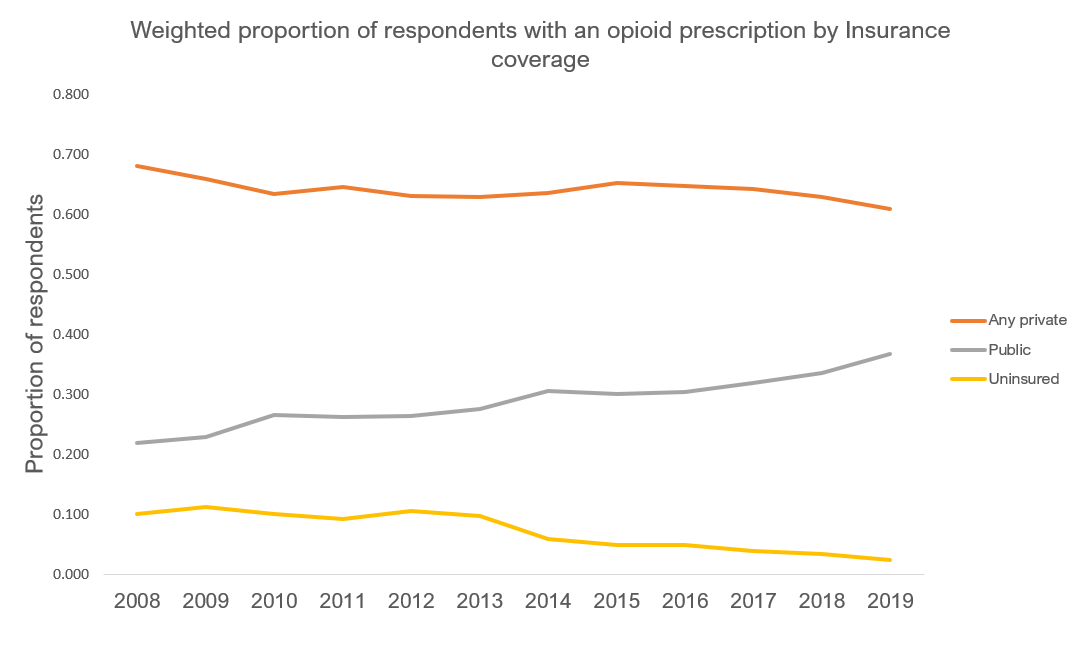


**Appendix 9C. Number/proportion of respondents with 1 or more prescription opioids by pain level.**

| Weighted totals | |  |  |  |  |  |  |
| --- | --- | --- | --- | --- | --- | --- | --- |
| Year | Not at all | A little | Moderate | Quite a bit | Extremely | Unknown | Total |
| 2008 | 611,929 | 577,207 | 369,546 | 435,023 | 237,626 | 96,197 | 2,327,528 |
| 2009 | 671,071 | 549,562 | 347,268 | 433,925 | 252,457 | 112,753 | 2,367,036 |
| 2010 | 639,709 | 546,303 | 387,092 | 446,148 | 265,046 | 119,603 | 2,403,900 |
| 2011 | 674,215 | 568,076 | 384,250 | 449,824 | 293,883 | 113,438 | 2,483,686 |
| 2012 | 586,028 | 528,811 | 434,385 | 497,510 | 275,535 | 79,019 | 2,401,288 |
| 2013 | 724,926 | 571,194 | 406,853 | 431,330 | 258,099 | 127,966 | 2,520,367 |
| 2014 | 730,814 | 544,739 | 402,414 | 504,092 | 242,009 | 197,107 | 2,621,176 |
| 2015 | 749,766 | 548,242 | 381,582 | 484,793 | 209,473 | 187,831 | 2,561,687 |
| 2016 | 609,593 | 453,194 | 313,709 | 376,276 | 194,393 | 168,661 | 2,115,825 |
| 2017 | 575,818 | 458,212 | 296,254 | 314,988 | 178,865 | 153,271 | 1,977,410 |
| 2018 | 545,378 | 438,623 | 277,763 | 301,047 | 161,885 | 171,356 | 1,896,052 |
| 2019 | 433,657 | 322,737 | 205,140 | 274,138 | 133,825 | 243,811 | 1,613,309 |


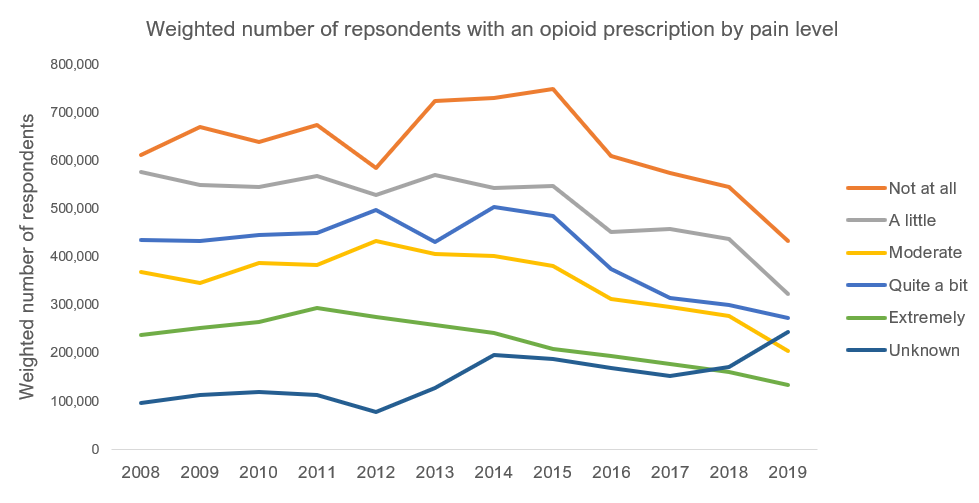


| Weighted proportions | | |  |  |  |  |
| --- | --- | --- | --- | --- | --- | --- |
| Year | Not at all | A little | Moderate | Quite a bit | Extremely | Unknown |
| 2008 | 0.263 | 0.248 | 0.159 | 0.187 | 0.102 | 0.041 |
| 2009 | 0.284 | 0.232 | 0.147 | 0.183 | 0.107 | 0.048 |
| 2010 | 0.266 | 0.227 | 0.161 | 0.186 | 0.110 | 0.050 |
| 2011 | 0.271 | 0.229 | 0.155 | 0.181 | 0.118 | 0.046 |
| 2012 | 0.244 | 0.220 | 0.181 | 0.207 | 0.115 | 0.033 |
| 2013 | 0.288 | 0.227 | 0.161 | 0.171 | 0.102 | 0.051 |
| 2014 | 0.279 | 0.208 | 0.154 | 0.192 | 0.092 | 0.075 |
| 2015 | 0.293 | 0.214 | 0.149 | 0.189 | 0.082 | 0.073 |
| 2016 | 0.288 | 0.214 | 0.148 | 0.178 | 0.092 | 0.080 |
| 2017 | 0.291 | 0.232 | 0.150 | 0.159 | 0.090 | 0.078 |
| 2018 | 0.288 | 0.231 | 0.146 | 0.159 | 0.085 | 0.090 |
| 2019 | 0.269 | 0.200 | 0.127 | 0.170 | 0.083 | 0.151 |


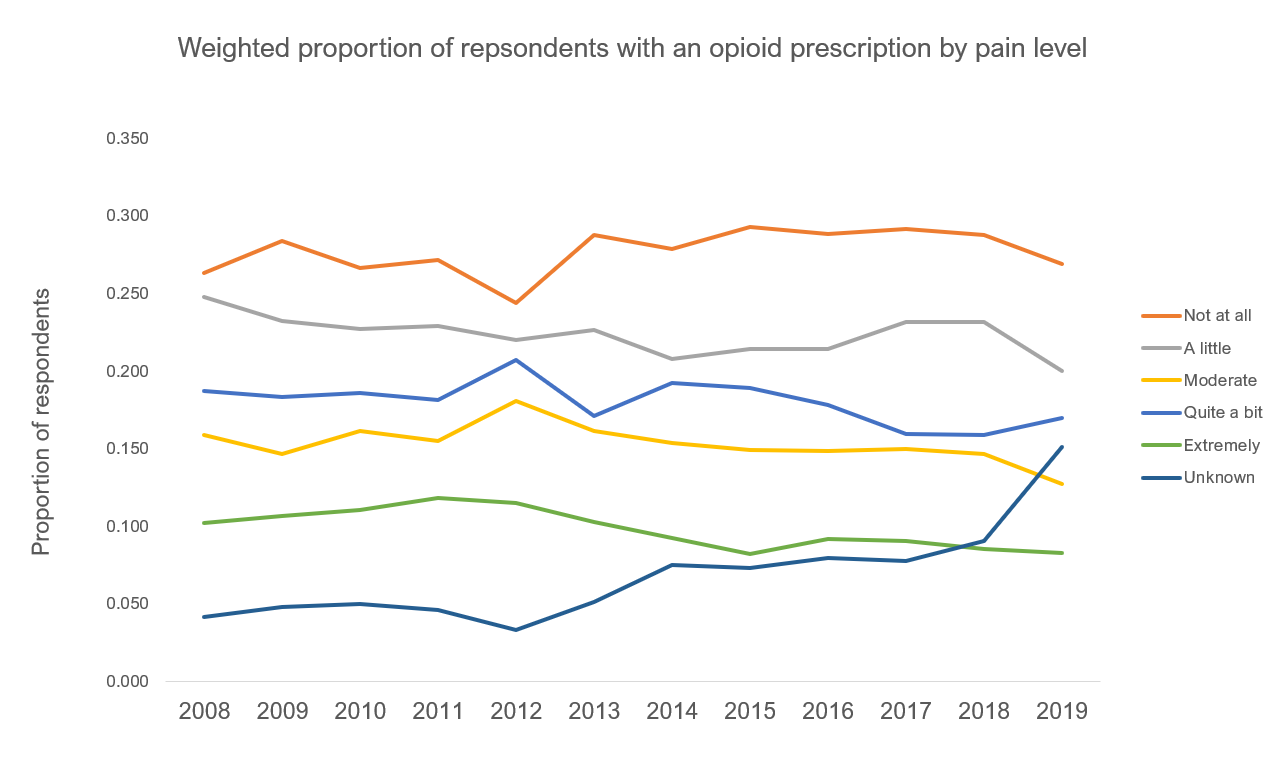


**Appendix 9D. Number/proportion of respondents who are Any (< 4 opioid prescriptions) and Frequent (4 or more opioid prescriptions) users of opioids.**

| Weighted totals |  |  |  |
| --- | --- | --- | --- |
| Year | <4 opioids | 4 or more opioids | Total |
| 2008 | 1,713,356 | 614,173 | 2,327,528 |
| 2009 | 1,738,927 | 628,109 | 2,367,036 |
| 2010 | 1,720,675 | 683,225 | 2,403,900 |
| 2011 | 1,761,648 | 722,038 | 2,483,686 |
| 2012 | 1,670,992 | 730,296 | 2,401,288 |
| 2013 | 1,795,210 | 725,157 | 2,520,367 |
| 2014 | 1,888,618 | 732,558 | 2,621,176 |
| 2015 | 1,901,304 | 660,383 | 2,561,687 |
| 2016 | 1,602,756 | 513,069 | 2,115,825 |
| 2017 | 1,380,525 | 596,885 | 1,977,410 |
| 2018 | 1,246,277 | 649,775 | 1,896,052 |
| 2019 | 1,130,283 | 483,025 | 1,613,309 |
|  |  |  |  |


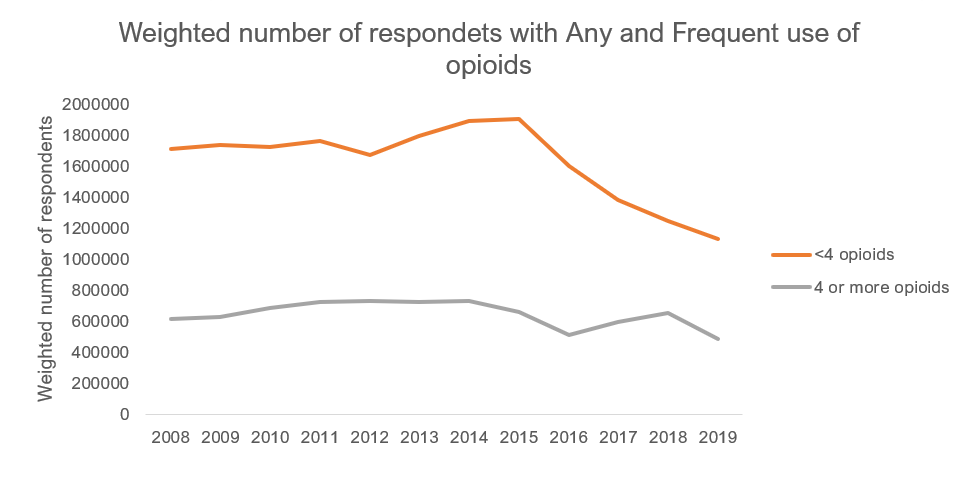


| Weighted proportion. | |  |
| --- | --- | --- |
| Year | <4 opioids | 4 or more opioids |
| 2008 | 0.736 | 0.264 |
| 2009 | 0.735 | 0.265 |
| 2010 | 0.716 | 0.284 |
| 2011 | 0.709 | 0.291 |
| 2012 | 0.696 | 0.304 |
| 2013 | 0.712 | 0.288 |
| 2014 | 0.721 | 0.279 |
| 2015 | 0.742 | 0.258 |
| 2016 | 0.758 | 0.242 |
| 2017 | 0.698 | 0.302 |
| 2018 | 0.657 | 0.343 |
| 2019 | 0.701 | 0.299 |


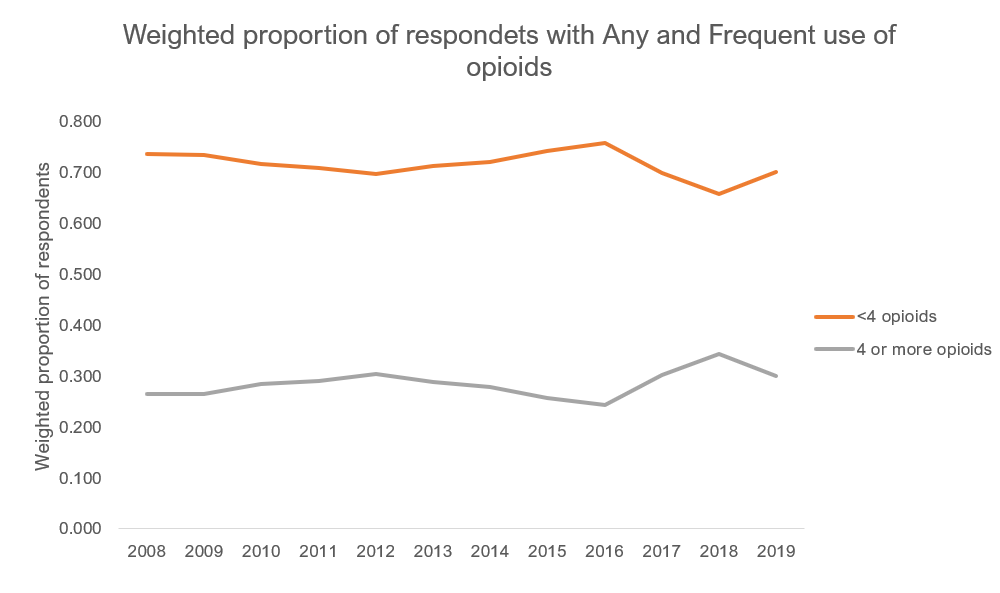


**Appendix 10. The average annual change in healthcare expenditure stratified by insurance type, opioid prescription filled, and pain level among respondents who had reporting filling an opioid prescription.**
